# randPedPCA: rapid approximation of principal components from large pedigrees

**DOI:** 10.1186/s12711-025-00994-y

**Published:** 2025-08-28

**Authors:** Hanbin Lee, Rosalind Françoise Craddock, Gregor Gorjanc, Hannes Becher

**Affiliations:** 1 Department of Statistics, University of Michigan, Ann Arbor, MI 48109 USA; 2 The Roslin Institute and Royal (Dick) School of Veterinary Studies, University of Edinburgh, Midlothian, EH25 9RG UK

## Abstract

**Background:**

Pedigrees continue to be extremely important in agriculture and conservation genetics, with the pedigrees of modern breeding programmes easily comprising millions of records. This size can make visualising the structure of such pedigrees challenging. Being graphs, pedigrees can be represented as matrices, including, most commonly, the additive (numerator) relationship matrix, $\varvec{A}$, and its inverse. With these matrices, the structure of pedigrees can then, in principle, be visualised via principal component analysis (PCA). However, the naive PCA of matrices for large pedigrees is challenging due to computational and memory constraints. Furthermore, computing a few leading principal components is usually sufficient for visualising the structure of a pedigree.

**Results:**

We present the open-access R package randPedPCA for rapid pedigree PCA using sparse matrices. Our rapid pedigree PCA builds on the fact that matrix-vector multiplications with the additive relationship matrix can be carried out implicitly using the extremely sparse inverse relationship factor, $\varvec{L}^{-1}$, which can be directly obtained from a given pedigree. We implemented two methods. Randomised singular value decomposition tends to be faster when very few principal components are requested, and Eigen decomposition via the RSpectra library tends to be faster when more principal components are of interest. On simulated data, our package delivers a speed-up greater than 10,000 times compared to naive PCA. It further enables analyses that are impossible with naive PCA. When only two principal components are desired, the randomised PCA method can half the running time required compared to RSpectra, which we demonstrate by analysing the pedigree of the UK Kennel Club registered Labrador Retriever population of almost 1.5 million individuals.

**Conclusions:**

The leading principal components of pedigree matrices can be efficiently obtained using randomised singular value decomposition and other methods. Scatter plots of these scores allow for intuitive visualisation of large pedigrees. For large pedigrees, this is considerably faster than rendering plots of a pedigree graph.

**Supplementary Information:**

The online version contains supplementary material available at 10.1186/s12711-025-00994-y.

## Background

Pedigrees continue to be extremely important in the fields of selective breeding and conservation genetics. Because pedigrees indicate the expected genetic similarity between individuals, they can be used to estimate the degree of inbreeding and coancestry, and the expected genetic merit of individuals when coupled with phenotype data. The pedigrees of long-established breeding programmes can easily contain millions of records. This sheer scale makes them difficult to visualise. One approach for visualising large pedigrees is to plot a pedigree graph with progeny groups collapsed into nodes [1]. This can be very effective, but it necessarily omits individuals from the visualisation.

There are numerous ways of representing a pedigree as a matrix. The most natural representation is perhaps the sparse adjacency matrix [2]. Alternatively, to encode the implied genetic relations between individuals, one can use the sparse adjacency matrix and, given a statistical model, derive the corresponding dense covariance (numerator relationship) matrix [3]. One popular tool for visualising matrices, and multidimensional data in general, is principal component analysis (PCA) [4, 5]. Technically, PCA is based on an Eigen decomposition of a covariance matrix or a singular value decomposition (SVD) of the underlying data matrix [6, 7]. For a symmetric data matrix, these two decompositions are closely related, as the Eigenvectors and singular vectors coincide, and the singular values correspond to the absolute values of the Eigenvalues. PCA produces a new matrix, whose columns are orthogonal linear combinations of columns in the data matrix [6, 7]. The columns are referred to as principal component scores, or simply principal components (PCs). Each PC accounts for a part of the variance shared across the columns of the data matrix, proportional to the respective Eigenvalues. The PCs are usually ordered by decreasing Eigenvalues. Thus, for many datasets, it is sufficient to obtain a few leading principal components to provide an overview of the structure of the data matrix.

Because pedigrees can be represented as matrices, they can also be visualised with PCA. However, few examples of pedigree PCA have been published [8–10], with a conspicuous absence of applications to large pedigrees. State-of-the-art genetics research is now based on highly informative genome-wide marker genotypes or whole-genome sequence data [11–14], with theory for understanding PCA in terms of genetic ancestry [15–17]. However, we still have large pedigrees, some with millions of individuals, which we would like to visualise efficiently. There are two key reasons for the absence of PCA for large pedigrees. First, the naive encoding of a pedigree as an additive relationship matrix (a dense covariance matrix) incurs quadratic storage complexity. Second, decomposing large dense matrices also has a significant compute complexity. For example, the SVD of a dense matrix has $O\left( min\left( nk^2, n^2k\right) \right)$ complexity for an $n \times k$ matrix, while the truncated SVD of a dense matrix has *O*(*nkr*) complexity to obtain the first *r* components [18]. There are also SVD algorithms that can operate on sparse matrices with *O*(*zk*) compute complexity for *z* non-zero elements in the matrix [19, 20].

Efficient methods for computing the Cholesky factor of the dense additive relationship matrix, and of its sparse inverse, the precision matrix, have long been known [21–23] . The Cholesky factor of the precision matrix is a function of a part of the sparse pedigree adjacency matrix and a vector of individuals’ Mendelian sampling variance, in line with the underlying quantitative genetic model [24–28]. The number of non-zero elements in the precision matrix and its Cholesky factor are therefore proportional to the number of individuals, enabling efficient linear algebra operations with large pedigrees [22, 26, 29–36].

Recent advances in randomised numerical linear algebra have led to the development of efficient algorithms for common matrix operations [37]. For example, randomised SVD can rapidly approximate a truncated SVD of a dense or sparse matrix, including matrices defined implicitly by matrix-vector multiplication [38, 39]. This randomised algorithm approximates the column space of a matrix using random test vectors and then computes the SVD on this low-dimensional approximation to obtain the dominant singular vectors and singular values. Despite the randomness of the algorithm, it behaves almost deterministically with high efficiency for matrices with rapidly decaying Eigenvalues. Critically, the algorithm only implicitly accesses the original matrix via matrix-vector products, without requiring the full matrix.

The aim of this contribution was to implement an efficient algorithm for rapid PCA of large pedigrees by using randomised SVD and the sparse Cholesky factor of the pedigree precision matrix. We achieved this by implicitly representing the matrix-vector products that are at the core of randomised SVD by solving a triangular system of equations based on the sparse pedigree precision Cholesky factor and the vector. The number of operations required for this operation is proportional to the number of individuals in the pedigree, hence scaling to millions of individuals. Another advantage is that the resulting algorithm has a low memory requirement and does not suffer from slow disk access, thus bypassing the significant computational bottlenecks faced by genotype PCAs. We benchmarked the algorithm with simulated data and a large empirical pedigree, and implemented it in the freely available randPedPCA R package. We also added functionality computing leading principal components from large sparse matrices using a Lanczos-based algorithm, implemented in the Spectra C++ library [40] and provided by the RSpectra R package [41]. Taken together, this work complements the existing toolbox for analysing large pedigrees.

## Implementation

The central piece of our rapid pedigree PCA is that we obtain the leading principal components of the additive relationship matrix $\varvec{A}$, while actually operating on $\varvec{L}^{-1}$, the Cholesky factor of the inverse of $\varvec{A}$ [21–23]. We developed code to obtain these principal components via randomised SVD [38] and also, more traditionally, via the RSpectra R package, which is an interface for the Spectra C++ library [40]. Following the pedigree quantitative genetic model [24–28], $\varvec{A}=\varvec{LL}^T$ is a covariance (coefficient) matrix, and $\varvec{L}$ is the Cholesky factor of $\varvec{A}$. Throughout this work, we refer to $\varvec{A}$ as the additive relationship matrix. But it is also known as the numerator relationship matrix, the relationship matrix, or covariance matrix. We approximate the decomposition of $\varvec{A}$ with a randomised SVD, which approximates the ‘structure’ of $\varvec{A}$ by creating an orthonormal range matrix, $\varvec{Q}$, with a reduced number of columns [38]. Again, instead of operating on $\varvec{A}$, we use $\varvec{L}^{-1}$, which is sparse and triangular. The number of non-zero entries in $\varvec{L}^{-1}$ is linear with the size of the pedigree. Conveniently, this matrix can be directly computed from a pedigree [21–23], for example, using the pedigreeTools R package [42]. We thus never actually perform a Cholesky decomposition of $\varvec{A}$.

In the following, we describe the key algorithms that enable the randomised SVD of large pedigrees. These are:

i. indirect matrix-vector multiplication of $\varvec{A}$ with a vector,
ii. centring the implicit data matrix,
iii. randomised SVD for pedigree PCA, and
iv. trace estimation for total variance.

To efficiently multiply $\varvec{A}$ with a vector $\varvec{x}$, we exploit the sparsity of $\varvec{L}^{-1}$. This is based on a well-known result [31], which we describe here for completeness. Note that $\varvec{Ax} = \left( \varvec{LL}^T\right) \varvec{x} = \varvec{L}\left( \varvec{L}^T\varvec{x}\right) = \varvec{b}$. The multiplication on the right, $\varvec{L}^T\varvec{x} = \left( \left( \varvec{L}^T\right) ^{-1}\right) ^{-1}\varvec{x} = \varvec{y}$, is a backward substitution with $\left( \varvec{L}^{-1}\right) ^T$ in the system of equations $\left( \varvec{L}^{-1}\right) ^T\varvec{y} = \varvec{x}$, because $\left( \varvec{L}^{-1}\right) ^T$ is an upper triangular matrix. The remaining multiplication, $\varvec{Ly} = \left( \varvec{L}^{-1}\right) ^{-1}\varvec{y} = \varvec{b}$, is a forward substitution with $\varvec{L}^{-1}$ in the system of equations $\varvec{L}^{-1}\varvec{b} = \varvec{y}$. Both substitutions cost *O*(*n*) operations and are available in standard linear algebra libraries [7, 43]. For example, the spam R package [44, 45] provides efficient implementations of these operations for sparse matrices. We summarise this routine in Algorithm 1. In the context of randomised SVD, which we will soon cover in more detail, $\varvec{x}$ is a random vector. Instead of performing the matrix-vector multiplication repeatedly with different random vectors $\varvec{x}$, we carry out one pass with the test matrix $\varvec{\Omega }$, whose columns give different vectors $\varvec{x}$.

**Algorithm 1:**
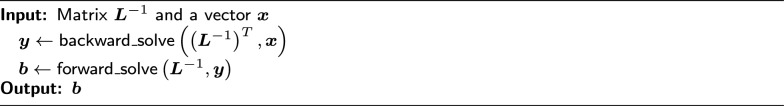
Efficiently multiplying the additive relationship matrix $\varvec{A}$ with a vector $\varvec{x}$ via the Cholesky factor, $\varvec{L}^{-1}$, of the precision matrix

Now, we show how to efficiently ‘centre’ the (pedigree) additive relationship matrix $\varvec{A}$ for PCA. $\varvec{A}$ is closely related to the genotype relationship matrix $\varvec{G}$, which is commonly used for PCA in genetics. The genotype relationship matrix is commonly calculated as $\varvec{G} \propto \widetilde{\varvec{X}}\widetilde{\varvec{X}}^T$, where $\varvec{X}$ is a genotype (data) matrix with loci in columns and individuals in rows and $\widetilde{\varvec{X}}$ is the column-centred genotype matrix [46]. Because $\varvec{G}$ is computed from a centred genotype matrix, we refer to it as ‘centred’, but note that its column means are non-zero. The genotype $x_{i,l}$ of individual *i* at locus *l* can be viewed as a random variable with respect to the meiotic process on a pedigree. For a given infinite pedigree, the expectation of $\varvec{G}$ according to the meiotic process is the ‘centred’ $\varvec{A}$:

1 $$\begin{aligned} \mathop {}\mathbb {E}[\varvec{G}|pedigree] = \widetilde{\varvec{A}} = \varvec{P}_n \varvec{A} \varvec{P}_n, \end{aligned}$$

where *n* is the number of individuals, $\varvec{P}_n = \varvec{I}_n - \frac{1}{n}\varvec{1}_n\varvec{1}_n^T$, and $\varvec{1}_n$ is a column vector of *n* ones. Hence, the PCAs of $\varvec{G}$ and $\widetilde{\varvec{A}}$ are closely connected, which we demonstrate in the Results section. Using the right-hand side of Eq. (1), we can efficiently and implicitly ‘centre’ $\varvec{A}$. Consider $\widetilde{\varvec{A}}\varvec{x} = \varvec{P}_n\varvec{AP}_n\varvec{x} = \varvec{P}_n\left( \varvec{LL}^T\right) \varvec{P}_n\varvec{x} = \varvec{P}_n\left( \varvec{L}\left( \varvec{L}^T\left( \varvec{P}_n\varvec{x}\right) \right) \right) = \widetilde{\varvec{b}}$. The right-hand multiplication of $\varvec{P}_n$ to a vector $\varvec{x}$ is simply subtracting the mean of the elements from the vector, that is, centring the vector, so we do not need to explicitly form $\varvec{P}_n$. This gives us Algorithm 2 for efficiently multiplying the ‘centred’ additive relationship matrix $\widetilde{\varvec{A}}$ with a vector $\varvec{x}$.

**Algorithm 2:**
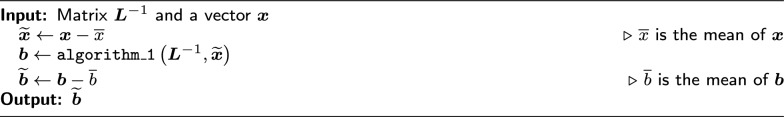
Efficiently multiplying the ‘centred’ additive relationship matrix $\widetilde{\varvec{A}}$ with a vector $\varvec{x}$ via the Cholesky factor, $\varvec{L}^{-1}$, of the precision matrix

With the essential matrix-vector multiplication algorithms in hand, we can establish the randomised SVD algorithm for rapid pedigree PCA following [38]. The algorithm multiplies $\varvec{A}$ to a random matrix of independent Gaussian variables $\varvec{\Omega } \in \mathbb {R}^{n \times l}$ via Algorithm 2, and applies QR decomposition (with $O\left( nl^2\right)$ compute complexity) to obtain an approximate rank-*l* orthogonal range matrix $\varvec{Q} \in \mathbb {R}^{n \times l}$. To increase accuracy, the integer *l* is chosen to be larger than the desired number of principal components, *k*. According to [38], there is no need to set *l* to more than 2*k*. This multiplication step can be repeated multiple times to improve the range matrix, where subsequent steps multiply $\varvec{A}$ to $\varvec{Q}$ from the previous step instead of $\varvec{\Omega }$. Once $\varvec{Q}$ is obtained, we compute $\varvec{B} = \varvec{Q}^T\varvec{A}$, see [38], efficiently via backward substitution with $\left( \varvec{L}^{-1}\right) ^T$. Given that the number of desired columns, *k*, in $\varvec{B}$ is $\ll n$, it is then possible to run an ordinary SVD, which returns: an $n \times l$ matrix of left singular vectors, $\varvec{U}$; an $l \times 1$ vector of singular values, $\varvec{d}$; and an $l \times l$ matrix of the right singular vectors, $\varvec{V}$. From each of these, we then use only the *k* first columns (or elements). The matrix-vector product $\varvec{QUd}^2$ gives the *k* approximate principal component scores of $\varvec{A}$ [6, 7]. While fully customisable, we have set default values of $k=10$ and $l=15$ in our implementation, which we found to work well on simulated and empirical data. We square the singular values because the entries of $\varvec{d}$ are the singular values of $\varvec{L}$ (due to using the backward substitution to compute $\varvec{B}$). The vector $\varvec{d}$ also represents the standard deviation of each approximated principal component. This randomised SVD is summarised in Algorithm 3.

**Algorithm 3:**
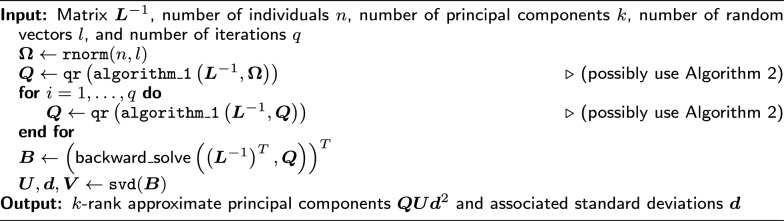
Approximate PCA of the additive relationship matrix $\varvec{A}$ (possibly ‘centred’) via the randomised SVD of the precision matrix’s Cholesky factor $\varvec{L}^{-1}$

PCA implementations usually return the principal component scores and associated standard deviations, which enables the reporting of the proportion of total variance captured by each principal component. The total variance is the sum of the squared standard deviations of the principal components, or equivalently the trace of covariance matrix. Because truncated SVD returns only a subset of the principal components, and associated standard deviations, the total variance and variance proportions cannot be computed. To calculate the total variance, we use the Meuwissen [29] algorithm, implemented in the pedigreeTools R package [42]. This computes all the individuals’ inbreeding coefficients. We then sum these and add *n* to the sum, all without forming the matrix $\varvec{A}$. When using the ‘centred’ additive relationship matrix $\widetilde{\varvec{A}}$, we efficiently compute its trace as $\textrm{Tr}(\varvec{A}) - \frac{1}{n} \varvec{1}^T \varvec{A} \varvec{1}$. We derived this expression by applying the trace operator to Eq. (1):

2 $$\begin{aligned} \begin{aligned} \textrm{Tr}(\varvec{P}_n \varvec{A} \varvec{P}_n)&= \textrm{Tr}(\varvec{A} \varvec{P}_n^2) \quad \because \textrm{Tr}(AB) = \textrm{Tr}(BA)\\&= \textrm{Tr}(\varvec{A} \varvec{P}_n) \quad \because \varvec{P}_n \text { is an idempotent matrix}\\&= \textrm{Tr}\left( \varvec{A} \left( \varvec{I}_n -\frac{1}{n}\textbf{1}_n \textbf{1}_n^T\right) \right) \quad \because \varvec{P}_n = \varvec{I}_n-\frac{1}{n}\textbf{1}_n\textbf{1}_n^T\\&= \textrm{Tr}(\varvec{A}) - \frac{1}{n}\textrm{Tr}( \varvec{A} \textbf{1}_n\textbf{1}_n^T) \\&= \textrm{Tr}(\varvec{A}) - \frac{1}{n} \textrm{Tr}(\textbf{1}_n^T \varvec{A} \textbf{1}_n) \quad \because \textrm{Tr}(AB) = \textrm{Tr}(BA)\\&= \textrm{Tr}(\varvec{A}) - \frac{1}{n} \textbf{1}_n^T \varvec{A} \textbf{1}_n \quad \because \textbf{1}_n^T \varvec{A} \textbf{1}_n \text { is a scalar} \end{aligned} \end{aligned}$$

The trace of the ‘centred’ additive relationship matrix thus depends on the trace of the non-centred version, which is straightforward to compute from the pedigree. If the user only has access to the $\varvec{L}^{-1}$ matrix, and not the pedigree, the trace of $\varvec{A}$ must be estimated. Inspired by the randomised SVD algorithm, we initially tested the popular Hutchnson algorithm [47], but we found that its estimate variance was too high for a computationally feasible number of random vectors. Thus, we implemented the recent quadratic improvement of this algorithm, which is called Hutch++ [48]. Similar to our randomised SVD implementation, this trace estimation algorithm evaluates matrix-vector products of $\varvec{A}$ with a random vector $\varvec{x}$, without actually computing $\varvec{A}$. Instead, an ‘oracle’ function is used (Algorithm 1) that returns the matrix-vector product $\varvec{Ax}$ by working with the sparse Cholesky factor $\varvec{L}^{-1}$, see Algorithm 1. Our implementation of Hutch++ also allows for implicit centring via Algorithm 2.

We have implemented the above algorithms in the open-source randPedPCA R package. The package also contains utility methods extending R’s summary and print functions for pedigree PCA objects, and a function for 3D plots with projections that utilises the optional dependency rgl. Additionally, the package contains documentation for all exported functions, it includes three documented example datasets (two of which we describe in the Results section), a vignette, and a test suite to minimise inadvertent changes during development.

## Results

To demonstrate the utility of, and to help build an intuition about, pedigree PCA, we ran randPedPCA on simulated data and on a large Labrador Retriever pedigree. We also compared the wall clock time of randPedPCA’s randomised SVD and RSpectra methods to that of a naive PCA implementation.

### Simulated data

We generated synthetic pedigree and genotype data for two scenarios, ‘2pop’ and ‘4pop’, using the forward-in-time simulator AlphaSimR [49]. For the 2pop scenario, we created two populations, 1 and 2, of 50 individuals each, using coalescent simulation. These populations originated from the same ancestral population 100 generations ago. We saved 11,000 segregating loci across 10 chromosomes. We then added two traits, each with genetic variance of 1.0, and a negative genetic correlation of − 0.3 The environmental variance of each trait was set to 2.0 and covariance was set to 0.0. Both traits had 100 causal variants per chromosome. We then selected for trait 1 in population 1 and for trait 2 in population 2, using the top ten individuals for the respective trait, and generating 50 offspring in each population per generation. We maintained this regime for 20 additional generations. In generation 10, we created a hybrid/crossbred population of 50 individuals, using parents from 1 and 2, which had been differentially selected for traits 1 and 2, as described above. Each subsequent generation of the hybrid population had 50 individuals. To generate these, we chose two parents from each, 1 and 2, and six from the hybrid population. From each population, we selected the individuals that scored the highest in a selection index that weighted both traits equally.

In the 4pop scenario, we did not apply selection. We started from one panmictic ancestral population of 200 individuals, again generated via coalescent simulation, which we propagated for 10 discrete generations at constant size. After that, we split this ancestral population into four daughter populations of 50 individuals each, without gene flow between them. We then propagated each for nine generations, again at a constant population size, and recorded pedigree and SNP genotypes at neutral markers.

For both scenarios, we ran pedigree PCA with randPedPCA. We carried out genotype PCA using R’s built-in prcomp function (with centring) on all 11,000 loci.

#### 2pop scenario

**Fig. 1:**
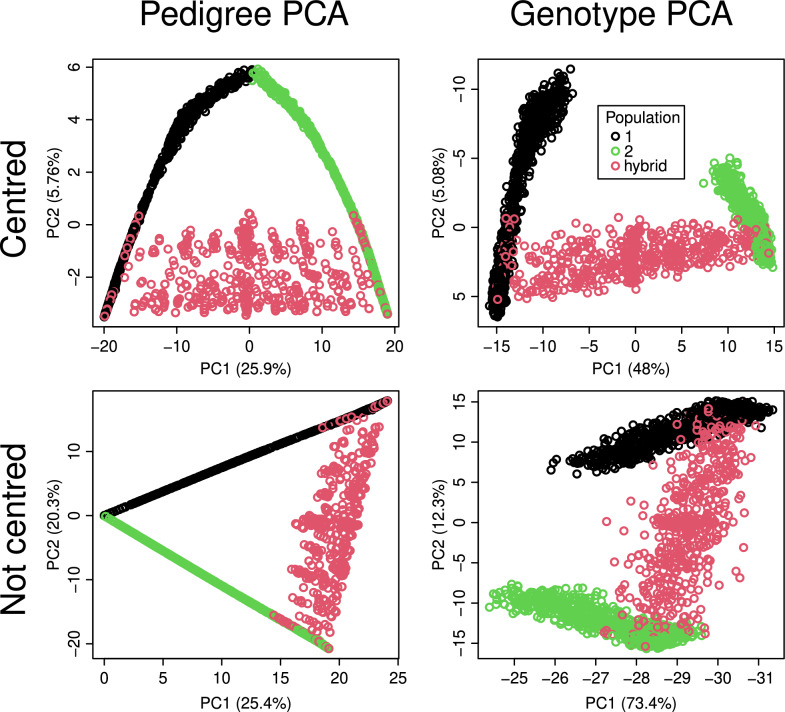
Scatter plots of the first two principal components computed from the pedigree and SNP genotypes of the 2pop scenario with centring (top row) or without centring (bottom row). The plots on the left were generated with randPedPCA and thus show approximate scores and percentage of captured variance. We ran the standard PCA on all SNP markers. The legend applies to all panels.

All PCAs of the 2pop scenario (Fig. 1) showed a rapid decay of the variance captured by the principal components, with principal component 3 always capturing less than 3% of the total variance. There was a clear difference between PCA on non-centred versus centred data. For the non-centred data, see bottom panels of Fig. 1, PC2 captured a comparatively high proportion of the total variance and separated populations 1 and 2. PC2 explained 20% of the variance for the pedigree and 12% for the SNP genotypes. PC1 aligned well with time, that is, generation number of the simulated populations. The Spearman correlation coefficient, $\rho$, between generation number and PC1 was 0.88 for the pedigree data and − 0.90 for the SNP data. PC1 captured a large amount of the total variance, 25% in the case of the pedigree PCA and 73% for the genotype PCA.

With centring (Fig. 1, top panels), there was a greater drop in the variance captured between PC1 and PC2. For the PCAs on the centred data, it was PC1 that separated populations 1 and 2. It captured 26% of the total variance for the pedigree PCA and 48% for the genotype PCA. PC2 captured considerably less of the total variance, 5.7% for the pedigree PCA and 5.1% for the genotype PCA. With centring, it was PC2 that aligned with the generation number, with $\rho$ values of -0.97 for the pedigree PCA and 0.86 for the genotype PCA. The top-right panel of Fig. 1 shows how the generations of population 1 (in black) align with PC2. The generation of population 2 (in green), also spread out along PC3 (not shown). Comparing the plots of pedigree PCA with those of genotype PCA (the left and right-hand panels of Fig. 1), there was a strong resemblance in general shape. The axes were flipped in both cases, as PCA does not preserve the sign of individual principal component score vectors. The most notable difference between plots of the pedigree and the genotype PCA was that the individuals at the top of the pedigree, which are the founders of populations 1 and 2, are shown at the same point in the pedigree PCA plot, despite being genetically diverged. The genotype PCA plots on the right-hand side of Fig. 1, however, show populations 1 and 2 as separate.

#### 4pop scenario

**Fig. 2:**
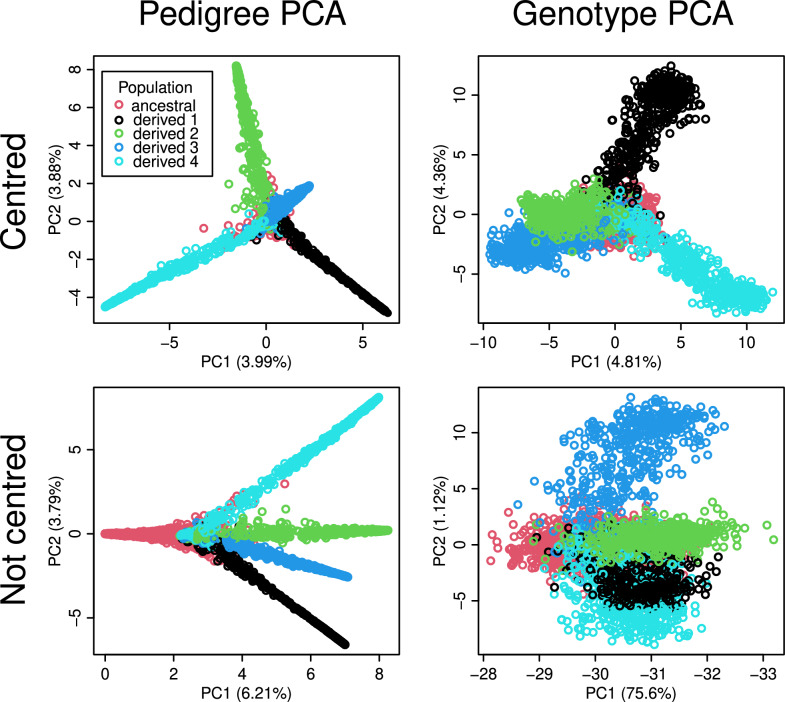
Scatter plots of the first two principal components computed from the pedigree and SNP genotypes of the 4pop scenario with centring (top row) or without centring (bottom row). The plots on the left were generated with randPedPCA and thus show approximate scores and percentage of captured variance. We ran the standard PCA on all genotype markers. The legend applies to all panels

The PCAs of the 4pop scenario (Fig. 2), had variance components that decayed more slowly. Again, without centring (bottom panels), PC1 aligned well with the generation number of the simulated individuals, capturing 6.2% of the total variance for the pedigree and 76% for the SNP genotypes. With centring (top panels), it was the population structure that dominated the top principal components, whereas time aligned only with PC4 (not shown). For both pedigree and SNP genotypes, patterns emerged that resembled a central point cloud (red dots) with four spikes (green, light blue, dark blue, and black dots). The spike ends were arranged like the points of a regular tetrahedron when also taking into account PC3 as the third dimension (not shown). The central cloud contained individuals of the ancestral population, whereas each spike corresponded to one derived population. We observed similar-sized variance components of approximately 4 to 5% for principal components 1 and 2 for both the genotype and the pedigree PCA with centring.

### Labrador Retriever data

To demonstrate the application of randPedPCA to empirical data, we used the registered canine pedigree of the Labrador Retriever, provided by the Kennel Club, UK. As the most popular breed in the UK, the Labrador Retriever pedigree consists of 1,486,764 records covering 70 years (1955 to 2025). However, because records were not fully digitised until 1990, the pedigree is incomplete, with founders occurring throughout the pedigree. We analysed it as such, without defining unknown parent groups. Data cleaning was minimal, with the addition of individual records for any sires and dams not listed as individuals, and the resolution of two pedigree loops (where an individual is found to be their own ancestor). We identified these loops when sorting the pedigree, and located both offending individuals using the visPedigree R package [50]. Both pedigree loops had been caused by incorrect sire allocation, which we set to missing. We then reordered the pedigree from the ancestors to the descendants. Generations were counted forward in time, where generation one represents the founders. All non-standard recorded coat colours were set to unknown to compare population structure only for black, yellow, and chocolate coat colours. Finally, we performed a centred pedigree PCA, using randPedPCA.

**Fig. 3:**
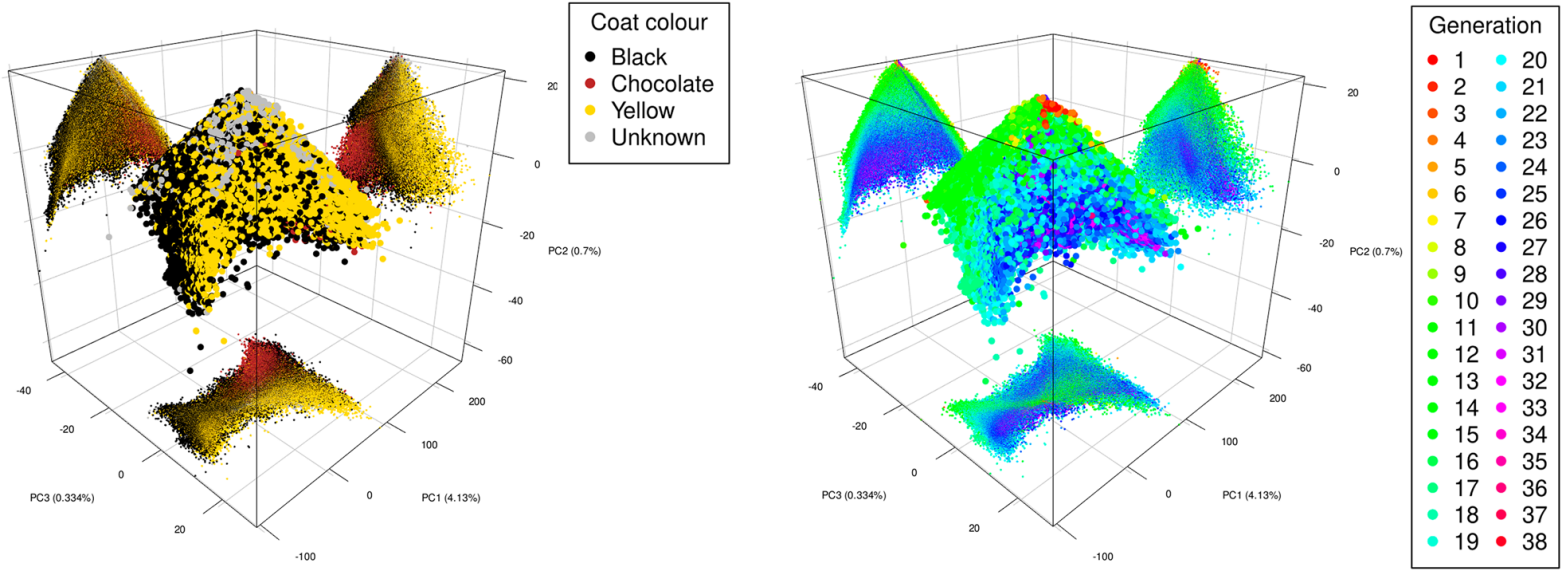
3D scatter plots of the first three principle components for the UK Kennel Club’s Labrador Pedigree from 1955 to 2024. The left-hand plot highlights coat colour, while the right-hand plot highlights generation. Projections onto coordinate planes are provided with the same colour used in the main plots.

The first three principal components of the Labrador pedigree (Fig. 3) captured 6.5% of the total variance, with PC1 capturing 4.1%, PC2 0.70%, and PC3 0.33%. This aligns with a genotype PCA on a representative sample of Kennel Club registered Labradors [51]. PC2 was negatively correlated with time, whether measured by generation ($\rho =-0.63$) or year of birth ($\rho = -0.58$), see the right-hand panel of Fig. 3, vertical axis. We found an observable grouping of chocolate Labrador Retrievers, shown in brown in the left-hand panel of Fig. 3. The finding resonates with other studies that have reported differences between chocolate and non-chocolate (yellow and black) Labradors, e.g. [51, 52]. Although Labrador Retrievers were recognised as a breed by the Kennel Club in 1903, the chocolate colour was only included in 1930, partly due to the rarity of the chocolate colour. Only recently has their popularity increased. Thus, it is plausible that these show comparatively low scores on PC2, which is negatively correlated with time.

### Performance

To assess the performance of our package, we computed the two leading principal components for the simulated 2pop dataset and the Labrador Retriever pedigree. We compared the wall clock times of the randomised SVD and RSpectra-based methods of randPedPCA to R’s built-in prcomp on an Intel(R) Core(TM) i5-7500T laptop running Ubuntu 24.04 and R 4.5, running on one CPU core. For the 2pop dataset, randPedPCA computed an approximate PCA in 2.8 ms, while R’s naive prcomp took about 35 s, resulting in a time ratio of greater than four orders of magnitude (Table 1). We did not attempt to obtain such a wall clock time ratio for the Labrador dataset, as prcomp would require the dense $\varvec{L}$ matrix as input, which itself is prohibitively costly to compute and store from a pedigree with almost 1.5 million individuals. However, our randPedPCA randomised SVD implementation finished in under one second. For these benchmarks, we did not centre; as input, we used $\varvec{L}^{-1}$ for randPedPCA and $\varvec{L}$ for prcomp.

**Table 1:** Wall clock times for computing the two leading principal components without centring

	2pop	Labrador Retriever
Number of individuals	2650	1,486,793
Time for prcomp	35,051 ms	NA
Time for randPedPCA rSVD	2.8 ms	806 ms
Time for randPedPCA Spectra	4.1 ms	1219 ms

We used randPedPCA and R’s built-in prcomp on a standard laptop computer, running with one thread. The datasets are the 2pop simulation and the UK Labrador Retriever pedigree

When comparing the performance of randomised SVD and the Spectra approach, we found that the results were much affected by the number of principal components calculated and the computational environment. We also investigated the effect of centring. We always used $\varvec{L}^{-1}$ as the input. When centring, we also supplied an externally computed estimate of the total variance. When we computed only the two leading principal components, randomised SVD was always faster than the Spectra approach. With increasing numbers of principal components, Spectra tended to be faster. The switch happened between four and eight principal components. More details can be found in Supplementary file S1.

## Discussion

In this paper, we introduced a method for rapid PCA of large pedigrees, which complements the existing toolbox for analysing pedigrees, e.g. [1, 29–36, 53]. This is the pedigree counterpart to the popular genotype PCA [11–14]. This method is implemented in freely available randPedPCA R package. Below, we

i. describe the package’s user interface and performance,
ii. explore the meaning and impact of centring and how pedigree PCA compares to genotype PCA, and
iii. discuss incomplete pedigrees and applications.

### User interface and performance

Our R package for rapid pedigree PCA and visualisation, randPedPCA, is available from CRAN. The principal intended use is in Integrated Development Environments such as Rstudio or VSCode. But randPedPCA may also be used on the command line or non-interactively as part of pipelines, for instance, for quality control.

The package’s main user-facing function is called rppca. This function can take as input a pedigreeTools pedigree object or a sparse $\varvec{L}^{-1}$ matrix in spam format. It then computes the leading principal components. By default, this is done via randomised SVD. However, if method=’spec’ is set, then the leading principal components are computed via the Spectra C++ library. The function then returns an object of the S3 class rppca, which is modelled after R’s built-in prcomp class. We recommend that rppca is used with centring, that is, with the parameter center set to TRUE.

To inspect the outcome of the PCA, we created the S3 methods summary.rppca and plot.rppca. Because R’s base plotting is slow with many points, plot.rppca down-samples the number of dots to be shown to 10,000. This works through an index vector that is added to the rppca object by the function dspc. This function may also be run independently from plotting, providing more flexibility as to the number of dots shown and making plots reproducible.

For interactive 3D plotting, we generated a function called plot3DWithProj, which depends on the R package rgl. Because correctly installing rgl can be tedious, we declared it as a suggested package, not a formal dependency of randPedPCA. We believe that the 3D plots generated with plot3DWithProj are a very valuable data analysis tool as they allow one additional dimension to be concurrently inspected compared to a 2D plot. The common criticism of 3D plots, that it is not possible to know the correct position of a dot, is alleviated by the fact that we show 2D projections of all dots on to the coordinate planes, as shown in Fig. 3.

We showed that randPedPCA is approximately four orders of magnitude faster than a naive implementation when run on a simulated dataset of moderate size, 2650 individuals (Table 1). PCA on large pedigrees is virtually impossible without specialised approaches, and we are not aware of any previous large-scale out-of-the-box implementation. Thus, there is no other package to which we can compare randPedPCA in a fair way. An alternative to PCA is rendering a graph based on a pedigree’s additive relationship matrix, $\varvec{A}$, or the adjacency matrix, $\varvec{T}^{-1}$. This is also considerably slower than generating a PCA plot with randPedPCA. Our work is related to [1] who visualised large pedigree graphs by collapsing large progeny groups into nodes. Their approach retains the original display of a pedigree, while our approach projects pedigree information into lower dimensions. Our work is also related to [54] who developed a fast randomised PCA for sparse data and [55] who developed the rsvd R package for general matrices. But, we leverage the well-known result about the sparsity of the pedigree precision matrix and its Cholesky factor for a scalable PCA of large pedigrees. Lastly, our work is also related to scaling PCA to large genomic datasets, which are increasingly available in breeding programmes and biobanks. To this end [56–58] implemented randomised SVD, while [59] implemented probabilistic PCA.

### What is the data matrix in pedigree PCA and should it be centred?

PCA is usually performed on a data matrix that has features in columns and individuals in rows. It is often advised to centre the data matrix before PCA, see, for example, [6]. That is, the column means should be made equal to 0. One reason for this is that, when performing PCA on a dataset with non-zero column means, the first principal component tends to represent all column means’ differences from zero. PCA is usually carried out via

1. Eigen decomposition of a covariance matrix of the features or
2. SVD of the data matrix. Alternatively, PCA may also be carried out via
3. Eigen decomposition of the covariance matrix of the individuals.

This third approach is often not useful for ‘tall’ datasets with more individuals than features because it involves the decomposition of the large covariance matrix of individuals rather than the smaller covariance matrix of the features. The third approach is useful, however, for feature-rich ‘wide’ datasets such as omic data where there are often many more features than individuals, e.g. [60]. There is another key point where approach (3) differs from (1) and (2). Approaches (1) and (2) compute, among other things, a matrix of loadings, called the Eigen vectors or right singular vectors, depending on the method. The loadings matrix then has to be multiplied with the original data matrix to obtain the principal component scores. With approach (3), however, there is no need to go back to the data matrix. Rather, if we decompose the covariance matrix of individuals, the principal component scores are equal to the matrix of the left singular vectors, $\varvec{U}$, multiplied by the diagonal matrix of the squared singular values, $\textrm{diag}(d^2)$. This is also the approach we used when generating a pedigree PCA, based on implicitly decomposing the additive relationship matrix $\varvec{A}$, which is a covariance matrix of individuals.

Following the standard advice, genotype PCAs are usually based on centred allele dosages, although other implied statistical models and corresponding covariance matrices are also possible [61, 62]. Unlike for genomic data with individuals in rows and loci in columns, it is not immediately clear what is the corresponding data matrix for a pedigree and whether one should centre it for PCA. One can encode a pedigree graph as an adjacency matrix, though this representation does not imply a statistical model with a corresponding covariance matrix. For genomic data, the relationship matrix is the covariance matrix, $\widetilde{\varvec{X}}\widetilde{\varvec{X}}^T$, of the (centred) genotype data, $\widetilde{\varvec{X}}$. Hence, working back from the pedigree additive relationship matrix, a data matrix $\varvec{D}$ should fulfil the equation $\varvec{D}\varvec{D}^T=\varvec{A}$. Many matrices fulfil this criterion, one of them being $\varvec{L}$, which encodes the relations of each individual with all of its ancestors, following the pedigree quantitative genetic model [24–28]. This model treats the pedigree founders as a reference (base) population with additive genetic values distributed with mean zero and base population additive genetic variance. The non-founder values are then recursively modelled as a deviation from the average value of their parents, in line with recombination and segregation of the parental genomes. Hence, the pedigree founders serve as the centring reference point. However, the matrix $\varvec{L}$ could also be centred differently, depending on the aims of the analysis. While it is computationally costly to compute and store $\varvec{L}$ for large pedigrees, we have developed a scalable algorithm for such centring, which can also be used within pedigree PCA (Algorithm 2).

We found that plots of principal component scores obtained with and without centring commonly had swapped principal components 1 and 2. Without centring, principal component 1 tended to align with time or generation number, while accounting for a large amount of variance. With centring, time tended to covary with a lower-order principal component and population structure was reflected in the leading principal component(s). Centring also caused the plots of pedigree PCAs to look more like those of genotype PCAs, which are generally computed on a centred genotype matrix. Whether one centres or not and which individuals are chosen as the reference population, is related to one’s choice of the reference/mean point about which the covariance is defined and genetic relationships interpreted [61–64]. For example, [65] have recently used the realised identity-by-descent information from an ancestral recombination graph (pedigree gives expected identity-by-descent) and performed PCA with different time-depths to study how population structure changed over time. Our implementation centres to the mean across all individuals, but Algorithm 2 may, in principle, be adjusted to centre using a specific subset of reference individuals.

### Real world pedigrees and applications

One notable difference between the genotype and pedigree PCA plots was that for pedigree PCA all founding individuals were placed at the same location. This was obvious for the simulated 2pop scenario and the Labrador Retriever example. For both, the scores of the leading principal components were essentially identical for the founding individuals. This is plausible given the logic that PCA summarises common patterns in higher-order principal components. Pedigrees provide information about the expected provenance of the pedigreed individuals’ genetic material. The further ‘away’ from the founders an individual is located, the more information there is about that individual and its relationships to other individuals. However, there is no such information for individuals with no known parents, the founders of a pedigree. In the additive relationship matrix, the covariances between the founders are zero. Thus, for the sake of PCA, these individuals lack shared patterns that could be summarised. These individuals then contribute to low-order principal components, each to their own. But only the leading components are plotted as only those contain covariance patterns shared across many individuals.

This behaviour is not necessarily an issue, in particular when there are few founders which occur only in early generations. One straightforward fix would be to exclude any founder individuals from the PCA plot. If there are any external data on or assumptions about the genetic differentiation between founders [28, 66–68], the concept of metafounders [62, 63] may be used to group founders according to this information. For example, a pedigree with founders belonging to two (sub-)populations may be extended with two metafounders with appropriate covariance between them [63]. Pedigree PCA would then differentiate the founders between the two (sub-)populations. In this case, the metafounders, and not the founders, would end up with very similar scores in their leading principal components. Again, they could be omitted from the plot.

In addition to the visualisation of large pedigrees, our work will complement existing approaches to define and study sub-populations in breeding programmes [69–71]. It will also complement approaches to optimise selection of key individuals in conservation, genotyping, genome sequencing, and large-scale genomic estimation of breeding values, see, for example [60] and references therein. The logical extension of the approach we presented here is to combine pedigree and genotype data using the ‘single-step’ covariance matrix [72]. We have made initial progress towards this, leveraging the work of [73]. The full application is beyond the scope of the present paper.

## Conclusions

Visualising large pedigrees is a long-standing and challenging problem. Here we introduce the randPedPCA R package that rapidly computes the leading principal components from large pedigrees. This package thereby enables a straightforward and scaleable visualisation of large pedigrees. When such a PCA is combined with metadata, one can clearly study the structure of large pedigrees and highlight their key drivers of variation. The randPedPCA R package is freely available from CRAN and GitHub. In addition to the visualisation of large pedigrees, our work will complement existing approaches to define and study sub-populations in breeding programmes. It will also complement approaches to optimise selection of key individuals in conservation, genotyping, genome sequencing, and large-scale genomic estimation of breeding values.

## Supplementary Information

Supplementary Material 1.

## Data Availability

The randPedPCA R package is available from CRAN, which provides archived versions, too. The R code for the simulation of data and demonstration of the package is available on GitHub in the directory data-raw of the R project folder. The anonymised Labrador Retriever pedigree was provided by The Kennel Club. Project name: randPedPCA. Project home page: https://github.com/HighlanderLab/RandPedPCA. Archived version: https://CRAN.R-project.org/package=randPedPCA. Operating system(s): Platform independent. Programming language: R. Other requirements: The R packages RSpectra, pedigreeTools, and spam are installed automatically during setup. The package rgl is required for 3D plots. The dependence Matrix ships with R. License: GPL ≥ 2 Any restrictions to use by non-academics: None
